# Potential Clinical Use of CytoSorb^®^ for Ticagrelor and Rivaroxaban Elimination Prior to Emergency Orthopedic Surgery in Trauma Patients

**DOI:** 10.3390/life15071065

**Published:** 2025-07-03

**Authors:** Gabriele Melegari, Fabio Gazzotti, Federica Arturi, Elisabetta Bertellini, Andrea Tognù, Domenico Pietro Santonastaso, Matteo Villani, Francesca Coppi, Fabrizio Fattorini, Fabio Catani, Alberto Barbieri

**Affiliations:** 1 Department of Anaesthesia and Intensive Care, Azienda Ospedaliero Universitaria, Policlinico di Modena, Via Del pozzo 71, 41121 Modena, Italy; gazzottifabio@gmail.com; 2 School of Anaesthesia and Intensive Care, University of Modena and Reggio Emilia, 41125 Modena, Italy; federica.arturi.94@gmail.com (F.A.); alberto.barbieri@unimore.it (A.B.); 3 School of Medicine, University of Modena and Reggio Emilia, 41125 Modena, Italy; elisabettab53@gmail.com; 4 Department of Anaesthesia and Intensive Care, Gaetano Pini Hospital, 20122 Milano, Italy; tognu@msn.com; 5 Department of Anaesthesia and Intensive Care, Bufalini Hospital, Azienda USL Romagna, 47521 Cesena, Italy; d_santonastaso@hotmail.com; 6 Department of Anaesthesia and Intensive Care, Azienda Ospedaliero Universitaria, Policlinico di Parma, 56126 Pisa, Italy; mvillani@live.it; 7 Department of Cardiology, Azienda Ospedaliero Universitaria, Policlinico di Modena, 41121 Modena, Italy; francesca.coppi@unimore.it; 8 Department of Anesthesia and Intensive Care, Ospedale San Sebastiano, 00044 Frascati, Italy; fabrizio.fattorini1956@gmail.com; 9 Department of Orthopedic Surgery, University of Modena and Reggio Emilia, Azienda Ospedaliero Universitaria, Policlinico di Modena, 41121 Modena, Italy; fcatani59@gmail.com

**Keywords:** CytoSorb^®^, trauma, ticagrelor, rivaroxoban, removal

## Abstract

Background: Major orthopedic trauma in patients receiving anticoagulants such as ticagrelor or rivaroxaban poses a significant perioperative challenge, particularly in emergency contexts where bleeding risks are heightened and specific reversal agents may be unavailable. CytoSorb^®^, a hemoadsorption device, has demonstrated efficacy in cardiac surgery for drug removal. Its potential application in trauma surgery remains unexplored. Objective: This protocol describes a prospective clinical investigation assessing the feasibility and safety of CytoSorb^®^ hemoadsorption for the preoperative removal of ticagrelor and rivaroxaban in trauma patients requiring urgent orthopedic surgery. Methods: The proposed intervention involves integrating CytoSorb^®^ into a dedicated extracorporeal circuit under normothermic conditions (37 °C) with a blood flow of 150–200 mL/min for 300 min. Serial plasma samples will be collected at predefined intervals (0, 30, 60, 120, 240, 300 min) and drug concentrations. The primary outcome is the pharmacokinetic profile of drug clearance. Secondary endpoints include procedural safety, bleeding complications, and the feasibility of timely surgery. Expected Impact: The study aims to provide real-world data on the practical integration of CytoSorb^®^ for anticoagulant removal in orthopedic trauma care, potentially facilitating earlier surgery and improving perioperative safety. Findings may inform future randomized trials and protocol standardization.

## 1. Introduction

Major orthopedic trauma in patients undergoing anticoagulant therapy represents an increasingly complex clinical challenge, particularly within an aging population characterized by polypharmacy and multiple comorbidities. The emergency management of patients with fractures or major orthopedic trauma presents a significant clinical challenge, particularly in elderly and frail individuals receiving anticoagulant therapy [1]. The concomitant use of anticoagulants and antiplatelet agents substantially elevates the risk of perioperative bleeding complications, especially in an urgent surgical setting where time constraints limit the opportunity for standard anticoagulation reversal protocols. The use of anticoagulants significantly increases the risk of perioperative hemorrhage, which is already elevated in this patient population [2]. In cardiac surgery, numerous studies have demonstrated the efficacy of removing anticoagulant drugs via extracorporeal circulation, leading to near-complete drug clearance within hours and reducing bleeding risk without significant adverse events. Recent clinical evidence highlights a significant incidence of major hemorrhagic events in this vulnerable cohort, necessitating the adoption of innovative, rapid-response strategies to improve perioperative safety and patient outcomes. In this context, extracorporeal hemoadsorption therapies such as CytoSorb^®^ are emerging as promising adjuncts to traditional management approaches. As a result, the European Society of Anaesthesiology and Intensive Care (ESAIC) has included the CytoSorb^®^ sorbent in its guidelines for the removal of ticagrelor and rivaroxaban during emergency cardiac procedures [3,4,5,6].

Recent studies suggest that purification techniques may have applications beyond cardiac surgery. Dalmastri et al. described a case involving a patient with acute kidney injury and bilateral hydroureteronephrosis [7]. This patient, who was anticoagulated with apixaban for atrial fibrillation, underwent hemofiltration with continuous renal replacement therapy (CRRT) and CytoSorb^®^. This approach enabled the patient to receive surgical treatment that would have otherwise been postponed due to the risk of hemorrhage. Similarly, Zappulo et al. reported a comparable case, further demonstrating the potential of this approach for enabling emergency surgical interventions [8].

In trauma patients requiring urgent orthopedic surgery, the presence of direct oral anticoagulants (DOACs) and antiplatelet agents such as Ticagrelor represents a significant challenge. Among these agents, ticagrelor is particularly problematic due to its potent antiplatelet effect, high plasma protein binding, and the current lack of a specific reversal agent. Unlike dabigatran or factor Xa inhibitors, which can potentially be reversed with idarucizumab or andexanet alfa, no antidote is yet available for ticagrelor in clinical practice. Furthermore, platelet transfusion has limited efficacy in reversing its effect. In emergency settings where the time leading up to surgery is critical, and conventional reversal options are inadequate or unavailable, CytoSorb^®^ hemoadsorption offers a promising alternative, with early data and case reports supporting its capacity to remove ticagrelor from circulation. This study aims to evaluate the potential clinical use of CytoSorb^®^ to reduce bleeding risk and avoid surgical delay in trauma patients receiving Ticagrelor and/or Rivaroxaban. Our project aims to evaluate its efficacy and safety in orthopedic surgery for patients receiving ticagrelor and rivaroxaban. The primary objective is to assess the feasibility of reducing perioperative hemorrhagic complications and enabling more timely surgical interventions. The latest guidelines from the European Society of Regional Anaesthesia (ESRA) state that a washout period of 72 h is required for rivaroxaban, and five days is required for ticagrelor, before safely performing neuraxial anesthesia or deep nerve blocks [9,10].

## 2. Materials and Methods

**Figure 1:**
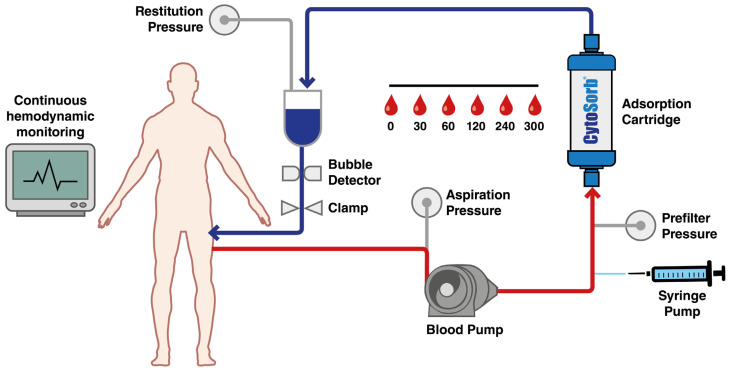
Haemofiltration treatment with CytoSorb^®^.

Patients with fractures or major orthopedic trauma requiring urgent surgery and who are currently taking ticagrelor or rivaroxaban are ideal candidates for CytoSorb^®^ therapy. The proposed clinical protocol incorporates the CytoSorb^®^ hemoadsorption device (CytoSorbents Corporation, Princeton, NJ, USA), USCytoSorb^®^ hemoadsorption cartridge CytoSorb^®^ hemoadsorption cartridges (CytoSorbents Corporation) into a dedicated extracorporeal circulation system, operating under normothermic conditions at 37 °C. The system maintains a blood flow rate of 150–200 mL/min over a total treatment duration of 300 min. Blood samples will be systematically collected at predefined time points and analyzed through highly sensitive liquid chromatography–tandem mass spectrometry techniques to accurately quantify plasma concentrations of ticagrelor and rivaroxaban. The entire procedure will be conducted under the strict supervision of trained specialists that are proficient in extracorporeal blood purification technologies. This methodology is currently applied in cardiac surgery settings. Blood withdrawal and reinfusion will be carried out through a central venous catheter (13–14 Fr), ensuring efficient circulation through the sorbent cartridge. Optimal clearance will be maintained with a pump-driven blood flow rate of 150–200 mL/min, in line with current best practices. Blood samples will be collected at 0, 30, 60, 120, 240, and 300 min to monitor plasma concentrations of ticagrelor and rivaroxaban. The use of this device in this way is authorized and codified. Comprehensive patient monitoring protocols will be implemented throughout the treatment to ensure procedural safety, including continuous hemodynamic assessment and the regular evaluation of coagulation parameters. The paper describes the project and revises the clinical rationale and management (Figure 1).

## 3. Application

The proposed treatment protocol involves 300 min of hemoperfusion before surgery to effectively remove anticoagulants and reduce the risk of intraoperative bleeding. During this process, healthcare providers will closely monitor patients for plasma anticoagulant levels, hemodynamic stability, and any complications related to perioperative bleeding. CytoSorb^®^’s ability to enhance hemostasis has important implications for anesthetic management. Given that these techniques are preferred for orthopedic surgery due to their less invasive nature compared to general anesthesia, CytoSorb^®^ may facilitate the timely execution of such regional anesthetic approaches.

## 4. Discussion

### 4.1. Management of Ticagrelor Suspension with Aspirin® and/or Aggrastat®

When managing patients who are receiving ticagrelor, it is important to balance the risk of thromboembolism with concerns about bleeding carefully. If ticagrelor needs to be stopped for emergency surgery, one option for bridging therapy is the short-acting glycoprotein IIb/IIIa inhibitor tirofiban (Aggrastat^®^) [11,12]. Tirofiban offers reversible platelet inhibition, providing short-term protection against thrombotic events while allowing for rapid clearance from the body within a few hours, thereby enhancing surgical safety. Aspirin is not absorbed by the CytoSorb® filter, allowing it to be maintained during surgery. This is especially important for patients at high risk for cardiovascular issues, as continuing aspirin therapy has been shown to reduce the risk of thrombotic complications during the perioperative period without significantly increasing the risk of major bleeding. The combination of aspirin maintenance and tirofiban bridging represents a viable strategy for mitigating thromboembolic risk while ensuring surgical feasibility [13].

### 4.2. Direct Oral Anticoagulants

Direct oral anticoagulants (DOACs), also known as novel oral anticoagulants (NOACs), have revolutionized anticoagulation therapy due to their predictable pharmacokinetics and lower risk of intracranial hemorrhage compared to vitamin K antagonists (VKAs) [14,15]. However, managing bleeding complications or urgent surgical procedures in patients on DOACs requires effective reversal strategies. In the last decade, several reversals have been proposed. For factor Xa inhibitors (apixaban, rivaroxaban, edoxaban), andexanet alfa (a recombinant modified human factor Xa decoy) has been approved as a specific reversal agent, demonstrating significant reductions in anti-factor Xa activity [16,17]. Alternatively, prothrombin complex concentrates (PCCs) may be used off-label when andexanet alfa is unavailable [18]. For dabigatran, a direct thrombin inhibitor, the specific reversal agent idarucizumab, a monoclonal antibody fragment, has shown the rapid and complete neutralization of anticoagulant effects [19]. Despite these advances, limitations exist, including high costs, restricted availability, and the need for further clinical data on the long-term outcomes of reversal strategies. Therefore, individualized patient management remains essential [20,21].

### 4.3. Mechanism of Adsorption in CytoSorb®

CytoSorb^®^ is a sterile, single-use medical device composed of porous polymeric polystyrene-divinylbenzene (PS-DVB) microspheres, suspended in a 150 mL saline solution. These microspheres are coated with polyvinylpyrrolidone (PVP), a highly biocompatible and hemocompatible material, allowing for direct use with whole blood without requiring plasma separation [22]. The porous structure of the microspheres forms a complex network of internal channels, creating an adsorption surface exceeding 40,000 m^2^. This unique architecture ensures high adsorption efficiency and prolonged performance, allowing continuous use for up to 24 h without requiring cartridge replacement. CytoSorb® is specifically designed to adsorb target molecules smaller than 55 kDa, ensuring uninterrupted treatment while maintaining optimal clearance capacity [23,24].

### 4.4. Extracorporeal Blood Purification Capabilities

CytoSorb^®^ is certified and indicated for the extracorporeal removal of multiple molecules directly from whole blood. It can be integrated into any extracorporeal circulation system to efficiently eliminate inflammatory mediators, including pro- and anti-inflammatory cytokines, bilirubin, in cases of liver dysfunction and hyperbilirubinemia, myoglobin, and to prevent acute kidney injury in rhabdomyolysis [25,26,27]. Furthermore, as mentioned above, it can remove Ticagrelor, a potent P2Y_12_ inhibitor used in cardiovascular patients, allowing for its rapid removal in urgent surgical settings, and rivaroxaban, a direct factor Xa inhibitor, enabling anticoagulation reversal in cases of severe bleeding or emergency interventions [5,6]. By combining high biocompatibility, broad adsorption capacity, and prolonged treatment duration, CytoSorb^®^ serves as an effective and versatile hemoadsorption therapy in critical care and extracorporeal blood purification. The adsorption process relies on multiple physicochemical interactions: hydrophobic interactions and Van der Waals forces. The polymer is inherently hydrophobic, which facilitates the adsorption of hydrophobic and amphipathic molecules. Non-covalent Van der Waals forces further stabilize the interaction between the target molecules and the polymer surface: electrostatic interactions, dipole–dipole forces, and weak electrostatic interactions occur between the sorbent’s surface and certain biomolecules, enhancing the retention of polar and charged compounds. These interactions contribute to the selectivity of the device in eliminating inflammatory mediators while preserving essential blood components. The controlled porosity of the polymer facilitates the selective entrapment of medium-sized molecules while allowing smaller molecules (e.g. electrolytes, small peptides) to pass through freely. Larger biomolecules, such as immunoglobulins and albumin, exceed the pore size threshold and are not adsorbed, ensuring minimal interference with physiological functions [28].

### 4.5. Adsorption of Ticagrelor and Rivaroxaban

CytoSorb^®^ has demonstrated the ability to efficiently adsorb certain lipophilic, protein-bound drugs, including ticagrelor and rivaroxaban, both of which have molecular weights within the effective adsorption range (~522 Da for ticagrelor and ~436 Da for rivaroxaban). Ticagrelor: this P2Y_12_ receptor antagonist is highly lipophilic and undergoes hepatic metabolism, with its active metabolite also contributing to platelet inhibition. Adsorption occurs primarily via hydrophobic interactions with the polymer surface, reducing systemic drug levels and potentially reversing its antiplatelet effect in critical bleeding situations. Rivaroxaban: as a direct factor Xa inhibitor, rivaroxaban is partially protein-bound (~90%) and demonstrates moderate hydrophobicity. It is effectively removed by CytoSorb through a combination of hydrophobic and π-π interactions with the aromatic structures of the polymer adsorbent, facilitating drug clearance and mitigating the anticoagulant effect in cases of life-threatening hemorrhage [29].

### 4.6. Fields of Application of CytoSorb®

CytoSorb® is indicated for the removal of various molecules from whole blood, enabling the treatment of critical conditions. Its applications include sepsis and septic shock through the removal of inflammatory cytokines; hepatic failure by eliminating bilirubin and ammonia; rhabdomyolysis by removing myoglobin; and the rapid elimination of anticoagulant drugs like ticagrelor and rivaroxaban. Sepsis and septic shock: sepsis is a leading cause of mortality in intensive care units, with septic shock pushing mortality rates above 80%. The “cytokine cascade” involves the uncontrolled release of pro- and anti-inflammatory cytokines, worsening the patient’s condition. CytoSorb^®^ is a valuable adjunctive therapy in sepsis, dramatically reducing inflammatory cytokines, stabilizing hemodynamics, decreasing the need for inotropic drugs, and reducing hospitalization time [30,31]. Recent studies emphasize the importance of early intervention, within 8–10 h of diagnosis. Other applications with severity: Beyond sepsis, CytoSorb^®^ is used in conditions characterized by cytokine storms, such as cardiac surgery, ECMO, severe burns, polytrauma, acute pancreatitis, severe influenza, meningitis [32]. Furthermore, CytoSorb^®^ improves liver function by removing bilirubin, bile acids, and ammonia—toxins that accumulate in the blood during hepatic insufficiency. It can support patients awaiting transplants or promote spontaneous liver regeneration. Compared to conventional dialysis, CytoSorb^®^ provides significantly more effective and immediate detoxification, simplifying its application within CRRT circuits and reducing complications. A different application is in the case of rhabdomyolysis: This condition leads to the massive release of myoglobin, which can result in acute kidney injury [33]. Traditional therapies are only partially effective, while CytoSorb has been proven to rapidly remove myoglobin, preventing renal damage, and speeding up renal function recovery [33]. It can be used as a preventive treatment or in conjunction with CRRT when renal failure occurs and liver dysfunction or organ recovery in donation after circulatory death [34,35,36]. Cardiac Surgery and removal of anticoagulant drugs: CytoSorb^®^ is highly effective at quickly removing Ticagrelor and Rivaroxaban, making emergency surgeries safer by reducing hemorrhagic risks. Studies have shown that the device removes approximately 99% of the drug in just two hours, significantly lowering intra- and postoperative complications. This efficiency has led to its inclusion in ESAIC guidelines. Additionally, CytoSorb^®^ helps manage the inflammatory response in cardiac surgery and ECMO, reducing vasoplegic shock, drug usage, and mortality.

### 4.7. Expected Outcomes

A significant reduction in the plasma concentrations of ticagrelor and rivaroxaban is expected within 240 min of CytoSorb^®^ hemoperfusion, allowing for surgery to be performed within five hours of starting treatment. This protocol aims to facilitate timely surgical interventions, thereby reducing the risk of perioperative hemorrhagic complications while maintaining hemodynamic stability. In addition, CytoSorb^®^’s selective adsorption capabilities help minimize the loss of essential blood components, improving hemostatic conditions and leading to better surgical outcomes. By decreasing the incidence of perioperative complications, the use of CytoSorb^®^ may shorten hospital stays, reduce healthcare costs, and increase the feasibility of emergency interventions across various surgical specialties, including neurosurgery and trauma surgery. Based on preliminary pharmacokinetic models and preclinical data, we anticipate a marked reduction in plasma levels of both ticagrelor and rivaroxaban within 240 min of initiating hemoadsorption therapy [29,37]. This decline is expected to create a safer window for surgical intervention, potentially enabling operative procedures to commence within approximately five hours of treatment onset. Moreover, the hypothetical extrapolation of the data suggests a favorable correlation between accelerated drug clearance and intraoperative hemodynamic stability, thereby reducing perioperative risks associated with residual anticoagulant activity. Secondary endpoints will include the evaluation of bleeding complications, transfusion requirements, and postoperative recovery trajectories.

### 4.8. Advantages and Disadvantages

The integration of CytoSorb^®^ hemoadsorption technology into clinical practice presents various advantages and disadvantages that need careful consideration to maximize its effectiveness. A key benefit is the notable decrease in the risk of perioperative bleeding. By effectively removing anticoagulants like ticagrelor and rivaroxaban from the bloodstream within a controlled time frame, CytoSorb^®^ helps reduce the risk of excessive bleeding during and after surgery. This is especially important for patients undergoing major orthopedic or trauma-related procedures. The ability to minimize bleeding is particularly valuable in situations where delays in surgical intervention could lead to serious complications. Expedited surgeries are crucial as they help lower the risks associated with prolonged immobilization, such as thromboembolism, infection, or the delayed healing of fractures [1,2,3,4]. One key advantage of CytoSorb^®^ is its potential to reduce the duration of hospital stays. By allowing faster and safer surgical interventions, CytoSorb^®^ may help decrease both preoperative waiting times and postoperative recovery periods. This can lead to cost savings for healthcare systems and improve patient flow in emergency departments and surgical units. Moreover, CytoSorb^®^’s ability to quickly clear anticoagulants enhances the use of regional anesthesia, which is often preferred for many orthopedic procedures because it poses a lower risk of systemic complications compared to general anesthesia. Regional anesthesia is associated with reduced rates of postoperative delirium, faster recovery of cognitive function, and fewer respiratory complications. Therefore, having access to CytoSorb^®^ could enable safer administration of neuraxial and peripheral nerve blocks in patients on anticoagulants, or in therapy with ticagrelor in line with the current guidelines from the good clinical practice recommended by the ESRA [9].

Despite its benefits, there are significant challenges associated with the implementation of CytoSorb^®^. One primary drawback is the need for specialized personnel to carry out hemofiltration sessions. This requires trained operators who can manage the extracorporeal circuits and monitor the patient’s hemodynamics throughout the procedure. Additionally, coordinating operating room schedules to accommodate hemoadsorption sessions adds another layer of complexity, especially in busy hospitals or during times of high patient influx. These logistical issues may limit their application in certain settings. CytoSorb^®^ offers several advantages over conventional pharmacological reversal agents, such as andexanet alfa and idarucizumab, particularly in emergency settings where immediate availability is critical. Unlike specific reversal agents that target only a single anticoagulant pathway, CytoSorb^®^ provides broad-spectrum adsorption capability, making it a versatile tool for patients on multiple antithrombotic medications. Nonetheless, before widespread adoption, careful consideration of economic factors and logistical integration into routine clinical workflows is essential. Cost–benefit analyses and resource allocation strategies should be developed to optimize the clinical utility of this technology. Furthermore, rigorous multicenter, randomized controlled trials are warranted to validate these preliminary findings, define standardized treatment protocols, and ensure reproducibility across diverse healthcare environments. Additional research should also explore patient-centered outcomes such as quality of life, the length of hospital stay, and long-term survival.

## 5. Conclusions

The integration of CytoSorb^®^ hemoadsorption therapy into the perioperative management of anticoagulated patients undergoing orthopedic trauma surgery represents a compelling advancement in the field of surgical critical care. By facilitating the rapid clearance of circulating anticoagulants, this approach has the potential to significantly mitigate bleeding risks, expedite surgical timelines, and enhance overall patient safety. This clinical protocol aims to explore the potential use of CytoSorb^®^ hemoadsorption for the perioperative reduction of ticagrelor and rivaroxaban levels in trauma patients undergoing urgent orthopedic surgery. While this approach may provide a novel option in scenarios where specific antidotes are unavailable or contraindicated, it involves an extracorporeal technique requiring central venous access, which introduces procedural risks. Therefore, any potential benefit in terms of drug clearance and surgical timing must be carefully weighed against these risks. The results of this study will provide preliminary real-world data on feasibility, pharmacokinetics, and may inform the design of future prospective interventional trials.

## Data Availability

The data presented in this study are available on request from the corresponding author.
